# Auditory verbal hallucinations result from combinatoric associations of multiple neural events

**DOI:** 10.3389/fnhum.2013.00239

**Published:** 2013-05-31

**Authors:** Massoud Stephane

**Affiliations:** Department of Psychiatry, Oregon Health and Science UniversityPortland, OR, USA

**Keywords:** AVH, schizophrenia, cognition, inner speech, language

## Abstract

While Auditory Verbal Hallucinations (AVH) refer to specific experiences shared by all subjects who have AVH—the perception of auditory speech without corresponding external stimuli, the characteristics of these experiences differ from one subject to another. These characteristics include aspects such as the location of AVH (inside or outside the head), the linguistic complexity of AVH (hearing words, sentences, or conversations), the range of content of AVH (repetitive or systematized content), and many other variables. In another word, AVH are phenomenologically heterogeneous experiences. After decades of research focused on a few explanatory mechanisms for AVH, it is apparent that none of these mechanisms alone explains the wide phenomenological range of AVH experiences. To date, our phenomenological understanding of AVH remains largely disjointed from our understanding of the mechanisms of AVH. For a cohesive understanding of AVH, I review the phenomenology and the cognitive and neural basis of AVH. This review indicates that the phenomenology of AVH is not a pointless curiosity. How a subject describes his AVH experiences could inform about the neural events that resulted in AVH. I suggest that a subject-specific combinatoric associations of different neural events result in AVH experiences phenomenologically diverse across subjects.

Decades of neuroscience research demonstrate that mental disorders are heterogeneous at the levels of neural circuits and genes (Insel, 2009), and that any specific defective neural circuit and any specific gene exists across diagnostic categories (Cross-Disorder Group of the Psychiatric Genomics Consortium et al., 2013). One could think that individual mental experiences/symptoms such as Auditory Verbal hallucinations (AVH) could offer better chance than mental disorders for corresponding to a neural specificity. However, like mental disorders, AVH are heterogeneous.

AVH are encountered in multiple psychiatric and neurological diseases and in non-clinical populations and are phenomenologically diverse. While all subjects with AVH necessarily share a common experience [the perception of speech in the auditory modality without corresponding external stimuli (Stephane et al., 2001a)], they differ from each other with respect to the characteristics of these experiences. For example, some subjects experience AVH inside their heads while others experience them outside their heads, and some subjects experience AVH with limited repetitive content while others experience AVH with variable rich content. A careful examination of these characteristics indicates that at least some of them correspond to neural specificities.

For years, research of AVH mechanisms has been largely based on the hypothesis that AVH result from a unitary deficit. However, emerging evidence suggest that a single deficit model does not explain the phenomenology of AVH and that multiple deficits are needed to adequately account for AVH experiences (Larøi and Woodward, 2007; Jones, 2010). To date, there are multiple models for the mechanisms of AVH; however, many of the existing models remain largely disjointed from AVH phenomenology and as such our understanding of AVH remains limited (Laroi et al., 2010).

For a cohesive phenomenological-neural understanding of AVH, here, I review the literature from the patient experience (phenomenology) to the brain (cognitive and neural basis). Based on current phenomenological and neural knowledge of AVH, I suggest an integrated phenomenological-neural framework.

## The phenomenology of AVH

Phenomenology as a philosophical discipline is a method for the study of first person (subjective) experiences for the purpose of identifying invariant inter-subjective (shared) phenomena “essences” (Zahavi, 2003). In a later development of philosophical phenomenology, heterophenomenology (Dennett, 1991), it was pointed out that shared first person subjective experiences are not infallible. It is commonplace for people, in certain situations, to experience movement and to see lines and color changes in the absence of movement, lines, or changing colors, respectively. In the tradition of heterophenomenology, first person subjective experiences are valid only as far as they are validated by natural science methodologies. Phenomenological research of AVH has proven to be in the latter tradition.

Phenomenological research of AVH shows that while all subjects with AVH share a common experience—hearing auditory speech without corresponding external stimuli, they differ from each other with respect to a number of characteristics of this common experience. For example, AVH differ across-subject in respect to space location (inside or outside the head), clarity (similarity to external speech or verbal thoughts), content (systematized or repetitive; new or previously experienced), linguistic complexity (hearing individual words, individual sentences, or conversations), concomitancy to normal external speech (hearing voices when alone in silence or while talking to other people), insight, or nosognosia [defined as awareness or not of the perceptions/object dissociation (Copolov et al., 2004)], gender (male or female voices), familiarity (familiar or unfamiliar voices), frequency, and loudness, and a number of other characteristics (Claude and Ey, 1932a,b; Jaspers, 1959; Sedman, 1966; Nayani and David, 1996; Stephane et al., 2003). In another word, AVH are phenomenologically heterogeneous experiences, which is to be distinguished from the heterphenomenological philosophical approach mentioned above (AVH phenomenological heterogeneity refers to experiences of AVH that differ across subject while philosophical heterophenomenology refers to the possibility of having erroneous first person subjective experiences and the need for validation of these experiences by natural science methods).

Phenomenological research of AVH has not been about marveling about the curiosity of AVH characteristics but, rather about the implications of these characteristics. For over a century, psychiatrists, psychologists, and philosophers considered that phenomenological variables point to qualitative (categorical) differences in hallucinatory experiences—that is, for example, AVH associated with AVH-anosognosia are qualitatively different from AVH with preserved nosognosia. As any given phenomenological variable is invariant only in a subset of subjects with AVH, they divided AVH, although inconsistently, into multiple sub-categories. For example, Claude and Ey (1932b) identified a “hallucinosis” subgroup when insight was present and a pseudohallucinations subgroup when the content was repetitive “etats obssessionels parasites” (Claude and Ey, 1932a). Jaspers, on the other hand used the term pseudohallucinations to refer to AVH that are concomitant to normal external stimuli, located in inner space, and lacked clarity of external perceptions (Jaspers, 1959). While Sedman considered pseudohallucinations to reflect AVH when insight into the unreality of the perception was present (lack of AVH-anosognosia) (Sedman, 1966). Jaspers also identified “sense-memory” subgroup when the content of AVH was previously experienced (Jaspers, 1959), a phenomenon also referred to as experiential hallucinations. Studies also examined AVH phenomenology for cues to discriminate between different categories of mental illness, and found that some AVH phenomenological variables differentiate between psychotic illnesses categories in use in that era (Lowe, 1973).

The above sub-categorization of AVH was considered as a premature closer; (Dening and Berrios, 1996) and, indeed, none of the above categories of AVH, beside experiential hallucinations, which could be produced by stimulation of the superior temporal gyrus (Penfield and Perot, 1963), were validated by neuroscience methods.

Motivated by numerous indirect evidence that the phenomenological variables of AVH reflect specific neural dysfunctions (see next section), our research group carried out a study to investigate the phenomenological space of AVH as a means to investigate the neural circuitry of AVH (Stephane et al., 2003). We used multidimensional scaling, a method that create an n-dimension maps based on distance or similarity data (here, the jacquard coefficient, which is the ratio of co-occurrence of two phenomenological variables to the sum of the occurrence of either), and found a three-dimension solution, linguistic complexity (hearing words, hearing sentences, hearing conversations), inner space-outer space locations, and self-other attribution of AVH. Based on evidence that language levels (lexical, sentence, discourse) are related to specialized neural resources (Caplan, 1992), that the neural correlates for sounds perceived in inner space differ from the neural correlates of sounds perceived in outer space (Hunter et al., 2003), and that of neural mechanisms for agency (self or other) (Feinberg, 1978), we suggested that the above dimensional structure mirrors the neural dysfunctions that result in AVH, such that AVH consisting of single words, sentences, or conversations result from dysfunction in lexical, sentence, and discourse neural resources, respectively. Similar arguments are made with respect to AVH experienced inside/outside the head and attributed to other/self in relation to neural resources for sound localization, and self-other distinction. Recently McCarthy-Jones et al. (2012) carried out cluster analysis using a wider subset of phenomenological variables than the one used in our previous study (Stephane et al., 2003) and found four clusters with main features that include repetitive content and running commentary, memory-like, replay of memories and nonverbal hallucinations. Both studies illustrate the useful information that could be derived from AVH phenomenology.

Furthermore, AVH phenomenology does not only provide a basis for the identification of categories of AVH that could be validated from neuroscience standpoint, it could also inform the experimental design in AVH research, (Larøi and Woodward, 2007; Laroi et al., 2010) an approach that has been proven fruitful (see last section).

The potential relevance of AVH phenomenology to the neural basis and treatment of AVH lead to another line of phenomenological research, the evaluation of the reliability of the patient report about hallucinations characteristics. Three decades ago, Junginger and Frame (1985) examined how consistent patients were in their report about a small subset of the phenomenological variables of AVH (such as frequency, loudness, location, and clarity). They asked patients to rate these variables on analog scales. Each variable was rated twice with question differently worded, and the consistency between the ensemble of questions pairs was computed. In our group, we undertook two approaches to examine the reliability of the report about AVH, the consistency of report and the frequency of endorsement of items. Both approaches are implemented in the computerized binary Scale of Auditory Speech Hallucinations (cbSASH) (Stephane et al., 2006a) The consistency approach is similar to that of Junginger and Frame however it examines the mismatches of binary variables of AVH. The frequency approach is based on standard techniques for detection of deception, where the endorsement of high numbers of infrequently endorsed statements indicates deception (Butcher et al., 1989).

The above considerations indicate that AVH phenomenology might not be an arbitrary collection of patients' first person subjective experiences; phenomenological research indicates the neural basis of AVH could be phenomenology-dependent. While indirect evidence in support of this thesis could be found in the literature for decades, it is not until recently that direct evidence of neural basis for AVH phenomenology was demonstrated.

## The neural basis of AVH phenomenology

### Indirect evidence of the neural basis of AVH phenomenology

The literature provides multiple indirect evidence of a neural basis for a number of phenomenological variables of AVH, including: AVH-anosognosia (unawareness of the perception-object dissociation), content (systematized or repetitive), space location (inner or outer space), and familiarity and the gender of the “voices.” Anosognosia of neurological symptoms (e.g., cortical blindness, and left side hemiparesis) is associated with symptom-specific neural correlates—lesions of the visual associative cortex (Magitot and Hartmann, 1926) or frontal lobes (McDaniel and McDaniel, 1991) in the case of cortical blindness, and lesions in the non-dominant motor cortex in the case of left side hemiplegia (Babinski, 1914). As AVH in the clinical population are symptoms of brain disease just like blindness or hemiplegia, AVH-anosognosia could be associated with specific neural substrates (Stephane et al., 2003).

It has also been shown that the neural correlates for speech perception differ according to whether the verbal stimuli are repetitive or variable (Cottraux et al., 1996), whether they are perceived to be inside or outside the head (Hunter et al., 2003), according to the familiarity of the speech sounds (Nakamura et al., 2001), and according to the gender of the perceived speech (Sokhi et al., 2005). While the difference in neural circuitry between the perception of actual feminine and masculine voices (for example) could result from different factors associated with these perceptions, it is plausible that similar differences in neural circuitry exist with the perception of hallucinated feminine and masculine voices. Consequently, it could be argued that the neural basis of AVH differs according to the content (repetitive or systematized), space location (inside or outside the head), and the gender and familiarity of perceived voices.

### Direct evidence of neural basis of AVH phenomenology

Recently, numerous studies have investigated the neural correlates associated with a number of the phenomenological variables of AVH and confirmed that the neural correlates of AVH are phenomenology-dependent (with respect to the investigated variables). One study has shown that, in patients with AVH, abnormalities in the right temporoparietal junction, a key area in the “where” auditory system, depend on the spatial location of the experience of AVH (inner or outer space) (Plaze et al., 2011). In another study, the loudness of AVH was associated with decreased activity in the bilateral angular gyrus, anterior cingulated gyrus, left inferior frontal gyri, and left temporal cortex (Vercammen et al., 2010). Furthermore, one fMRI study has shown that the acoustic clarity of AVH (similarity of AVH to speech or to thoughts), which was referred to as “sense of reality” in the study at hand, was associated with reduced language lateralization (Vercammen et al., 2010). However, another methodologically different fMRI study has shown that acoustic clarity was associated with activity in the inferior frontal gyri (Raij et al., 2009). The different findings could be related to differences in the methodology between the two studies (for example, in the first study, subjects were scanned while they performed a metrical stress evaluation task to activate inner speech, in the second study, patients were scanned during hallucinations). Therefore, while the exact neural correlates of the acoustic clarity of hallucinations remain unknown, these two studies provide evidence of a neural basis of acoustic clarity of AVH. Additionally, in drug naïve patients experiencing high linguistic complexity AVH (conversations), an FDG PET study showed higher metabolic rates in the left superior and middle temporal cortices, bilateral superior medial frontal cortex relative to psychotic patients without AVH (Horga et al., 2011). Furthermore, patients with AVH, compared to healthy controls, showed different patterns of speech related activation depending on the familiarity of speech (Zhang et al., 2008). While this does not indicate that patients with AVH consisting of familiar “voices” are different from patient with AVH consisting of unfamiliar voices, it emphasizes that dysfunction of neural processes for speech familiarity might play a role in the pathogenesis of AVH. Furthermore, preliminary evidence indicates that AVH with repetitive content respond to treatment by an antiobsessionalagent (Stephane et al., 2001b).

The studies outlined above, therefore, bring third person (objective) validation to the patients' first person subjective experiences of AVH. Whether such validation would extend to the other phenomenological variables is possible but remains to be proven.

## Cognitive models of brain activity associated with AVH

Three categories of cognitive models could be identified, including: inner speech, bottom-up and top-down processing of perceptions, and intrusions of thoughts and memories.

### Inner speech

Inner speech was a natural place to start in AVH research as this type of hallucinations refers to the perception of speech. In the mid twentieth century, based on observations that AVH are associated with lip movements without audible speech (Forrer, 1960), subvocal speech (SVS) that could be amplified and recorded (Gould, 1950), and Electromyographic speech muscle activity (Roberts et al., 1952), it was suggested that hallucinating patients are virtually hearing their self-generated faint SVS (Gould, 1948, 1950). This theory was short lived since maneuvers blocking SVS did not alleviate AVH (Stephane et al., 2001a). Given that both inner speech and AVH are associated with both motor and perceptual components (Sokolov, 1972; MacKay, 1992), We suggested, instead, that a disorder of generation of inner speech would result in a perceptual component (AVH) and a motor component (SVS) as an un-bothersome byproduct (Stephane et al., 2001a).

The vast majority of studies on this line of research focused on explaining the attribution to other of a self-generated inner speech. In the literature, inner speech theory is sometimes equated with a particular model (the forward model) (Frith and Done, 1988); here I discuss the ensemble of models that implicated inner speech in the pathogenesis of AVH.

The corollary discharge deficit or forward model is one of the most widely studied models of AVH mechanisms, and received empirical support from many studies (Ford et al., 2001; Stephane et al., 2006b). It was based on Feinberg theory that, “*motor commands in the nervous system are associated with neuronal discharges that alter activity in both sensory and motor pathways. They may act to inform sensory systems that the stimulation produced by movement is self-generated rather than environmentally produced. In this way these discharges are, at least in an abstract sense, crucial for the distinction of self and non-self and that could apply to higher functions*” (Feinberg, 1978). The forward model postulates that a disconnection between speech generation and speech perception results in a failure to compute the expected sensory experience of self-generated inner speech, and would lead to experiencing self-generated inner speech as alien.

Another model for self-other misattribution is that of altered preconscious planning of discourse, where a speaker generates a discourse that is incongruent with the goals or intentions of the speaker (Hoffman, 1986). While unintended tick (for example) is not attributed to other, Hoffman considered that the complexity of unintended inner speech, relative to unintended motor tick, suffices for the other-misattribution of the former (Hoffman, 1991).

Bentall (1990) argued that cognitive deficits such as the above do not explain the cultural, historical and emotional aspects of hallucinations “*hallucinators don't hallucinate random events*,” he suggested that self-other misattribution (defective reality monitoring) could result from a variety of deficits in metacognition (knowing that we know) as conceptualized by the American psychologist John H. Flavell (1979).

Fernyhough (2004), motivated by the infinite regress objection to the above theories, brilliantly examined the work of Vygotsky on inner speech development (Vygotsky, 1978) for clues about self-other misattribution. He suggested that alteration of the transformation that social speech undergoes to become inner speech (disruption of the internalization or re-expansion) could result in inner speech attributed to other (AVH), as inner speech is dialogical by nature.

Finally, in an imaging study carried out by our group (Stephane et al., 2006b), right handed hallucinating schizophrenia patient (but not matched non-hallucinating schizophrenia patients and healthy controls), showed abnormal laterality of the Supplementary Motor Area (SMA) activation during a speech generation task. As the SMA has been implicated in attributing self-generated actions to self, we suggested that the abnormal laterality of the SMA during the action of inner speech generation could result in occasional failure in attributing to self a self-generated speech (just like a right handed individual could fail carrying out actions with his/her left hand).

Unlike AVH, which could be experienced in outer space, inner speech is experienced in inner space. However, this apparent disorder in the spatial localization of inner speech received much less attention than self-other misattribution of inner speech. Nonetheless, The literature shows that three studies have examined this aspect of inner speech and all reported tendency of schizophrenia patients to confuse speech experienced in inner space with speech experienced in outer space (Harvey, 1985; Franck et al., 2000; Badcock, 2010; Stephane et al., 2010a).

### Bottom up and top down models

That perceptions depend on both external sensory stimuli and on representation of past perceptual experiences is an age-old idea. For example, Taylor (1979) observes that, in dim lighting, a rhomboid table could be perceived as rectangular table. Another example is that misspelled words in a text are often correctly read without noticing misspelling (Jaspers, 1959). Current AVH research implicates both the sensory pathway (bottom up) and past perceptual experiences and expectation (top down) in hallucinations pathogenesis.

Bottom-up factors point to unconstrained activity in the sensory and perceptual brain resources due to scarce external sensory stimuli. Auditory hallucinations have been often reported in patients with acquired deafness (Thewissen et al., 2005), in survivors of long solitary ordeals (Logan, 1993), and, to a lesser extent, during sensory deprivation experiments (Slade and Bentall, 1988). Furthermore, in psychotic patients, a dramatic social withdrawal preceding the onset of hallucinations has been reported (Hoffman, 2008).

Top down role in the pathogenesis of hallucinations has been also proposed for about half a century. French philosopher, Maurice Merleau-Ponty, considered hallucinations to reflect the “intentionality” of the hallucinator who creates a world according to his intentions and expectations (Merleau-Ponty, 2002), and Italian psychiatrist Silvano Arieti considered hallucinations to result from moments of heightened auditory attention “listening attitude” (Arieti, 1974). Recent studies bring support to these perspectives. Hallucinations scores were correlated with imagery-perception facilitation with pure tones (Aleman et al., 2003), and with the effect of semantic expectation on the perception of sentences (Vercammen and Aleman, 2010). Furthermore, increased incidence of auditory hallucinations is reported with high auditory attentional demands in healthy populations (Baraldi Knobel and Ganz Sanchez, 2009). It was suggested that an imbalance between top-down and bottom-up processing of stimuli could result in erroneous percepts that, when repetitive, would train the network to perceive hallucinations (Aleman et al., 2003). It was also suggested that a cognitive control deficit results in a failure of top-down inhibition of bottom-up erroneous percept (Hugdahl et al., 2009).

### Intrusions of thoughts and memories

Many studies in the past decade implicated intrusions of memories and thoughts in the pathogenesis of hallucinations. Based on findings indicating deficits in intentional inhibition in hallucinating patients, it was suggested that failure of suppression of irrelevant memories and other mental associations could result in intrusive memories experienced as hallucinations (Badcock et al., 2005; Waters et al., 2006). The theory finds support in many studies showing intrusions errors and false recognition with free recall and Sternberg paradigms (Brébion et al., 2007; Brebion et al., 2010), and that ruminations were related to hallucinations indirectly through the mediating factor intrusive thoughts (Jones and Fernyhough, 2009). Additionally, two fMRI studies showing deactivation of the parahippocampal gyrus prior to hallucinations (Hoffman et al., 2008; Diederen et al., 2010), and one magentoencephalography (MEG) study a decrease in theta-band power in the right hippocampus at the onset of AVH (van Lutterveld et al., 2012) bring further support to this theory. Studies also implicated intrusive thoughts in hallucinations through association with cognitive dissonance, the evidence, however, is not conclusive (van de Ven, 2012).

## Other aspect of brain activity associated with AVH

Some basic aspects of brain function affect all cognitive operations. These include, laterality, connectivity and the default modes system; all of which were implicated with AVH and schizophrenia in general. For example, studies have shown lack of right ear advantage in dichotic listening task (Hugdahl, 2009), and abnormal SMA laterality with a speech generation task in hallucinating schizophrenia patients (Stephane et al., 2006b). The above-mentioned forward model is a special case of dysconnectivity. Moreover, evidence of dysconncectivity implicates many other systems such as dorsolaterofrontal/superior temporal gyrus (Lawrie et al., 2002) and between the anterior cingulated gyrus and superior temporal gyrus (Mechelli et al., 2007), as well as dysconnectivity within the default mode network (van de Ven, 2012).

## Matches and mismatches between AVH phenomenology and AVH models

Here we take a close look on how the above models for AVH could explain some phenomenological variables but fail to explain others. This look is theoretical and based on current knowledge of AVH phenomenology and models. Direct empirical support is lacking as research designs did not generally take in consideration AVH phenomenology. It should be also mentioned that an exhaustive look on matches and mismatches may not serve much purpose and the account below is meant to be illustrative.

First, while any of the models of self-other misattribution could explain AVH attributed to other, they don't explain AVH attributed to self as encountered sometimes in schizophrenia (Stephane et al., 2003) and in survivors of long solitary ordeals who at some point during the ordeal undergo a transition from talking to themselves to experiencing their own voice as coming from outside (Logan, 1993). Furthermore, each of the self-other misattribution models may explain the self-other misattribution in certain phenomenological categories of AVH but not in others. For example, the forward model, presumes one speaker only “the hallucinator.” Therefore, it would account for self-other misattribution in AVH consisting of one, but not multiple “voices.” Altered discourse planning could produce unintended words and/or sentences (verbal messages) that result in a fragmented discourse. This model presumes that the unintended verbal messages are experienced as hallucinations and as such this model would explain self-other misattribution when AVH consist of words and sentences but not conversations. Finally Fernyhough's model presumes internal dialog between the hallucinator and other(s). Therefore, it could account for self-other misattribution when AVH consist of one or more “voices,” but not when AVH consist of multiple “voices” talking to each other. These arguments favor the visionary suggestion of Bentall, that self-other misattribution could result from a number of different deficits in the metacognitive domain “*It should be noted that many different types of cues are likely to be important in reality discrimination and that many different traits and deficits are therefore likely to be associated with hallucinatory experiences. The failure of reality discrimination in hallucinating patients might therefore be considered a final common pathway underlying their experiences, rather than the ultimate cause of their hallucinations. Moreover, it is probable that different kinds of cognitive deficits will be associated with different types of hallucinations*” (Bentall, 1990).

Additionally, as many times pointed out (Badcock, 2010; Jones, 2010; Laroi et al., 2010) self-other misattribution models do not account for aspect of AVH such as why AVH are repetitive in some and systematized in others, or why some subjects are aware of the lack of object for AVH while others are not (anosognosia). AVH “*are more than just words; it involves the perception of information about speaker identity and vocal affect*…,” Badcock notes (Badcock, 2010).

Similar reasoning could apply to Bottom up/top down and intrusions models. For example, heightened semantic expectation and listening attitude (top down) model could explain AVH consisting of words or sentences, but not conversations. Intrusive memories and intrusive thoughts could account for AVH with certain characteristics but not with others. Intrusions usually refer to singular events (the event, here, being a hallucination). When the hallucinations are repetitive words or sentences, it could be easily conceived that the hallucinations result from intrusions. However, when the hallucinations consist of complex multi-element auditory objects such as familiar voices issuing tirades of abuse untypical of the speaker (to whom the voices are attributed), or conversations with variable rich content (systematized), intrusions may not explains these hallucinatory experiences as well. Furthermore, metacognitive beliefs related cognitive dissonance does not account for AVH with positive content. Finally, inner speech, intrusive memories and intrusive thoughts are all experienced in inner space, and, as such, don't account for the outer space location of AVH. Of the above models, only models involving deficits in the inner space-outer space distinction, the “where” and “what” pathways, and top-down/bottom-up interaction could explain the outer space location of AVH.

## Combinatorics: resolving the phenomenological puzzle

Thus far it appears that AVH experiences are associated with rather complex patterns of commonality and differences across subjects, and that combinatoric association of AVH phenomenological variables accounts nicely for the observed phenomenological heterogeneity. Taking in considerations that at least some of the phenomenological variables appear to point to specific deficits in brain function, and that any given single deficit model does not adequately account for AVH, it could be concluded that the diverse AVH phenomenology results from combinatoric association of neural deficits. I suggest that AVH experiences require necessarily activity in Wernicke's area, which constitute a final common pathway (Stephane et al., 2000) for a widely distributed network that underlies emotions and aspects of cognition such as language, attention, memory. AVH arise from dysfunction (e.g., abnormal laterality or dysconnectivity) at combinations of nodes in the network that are subject specific, which determine the phenomenology of AVH in the subject at hand.

Concerning normal and abnormal brain function, combinatorics is not a novel idea. A century-old visionary theory of Korbinian Brodmann suggested that higher brain functions depend on a set of elementary neural resources combined in temporospatial patterns specific to each function (Brodmann, 1909); and this view is currently readily accepted in the neuroscience community (Stephane et al., 2010b). More importantly, emerging evidence about a small subset of AVH phenomenological variables supports this approach. For example, Waters and colleagues have shown that AVH could result from a combined deficits in intentional inhibition and context memory (Waters et al., 2006). The first would result in intrusions of memories into consciousness and the second would explain the other-misattribution of these memories. Additionally, based on imaging findings in our research group, we previously suggested that combined abnormal activity of Wernicke's area and of the SMA results in AVH; the former would explain the perceptual experience “Hearing” and the latter the other-misattribution of what is heard (Stephane et al., 2006b).

To date, the most substantiated combinatoric of deficits is that between self-other, and inner space-outer space confusions. Based on the phenomenological structure of AVH (Stephane et al., 2003), Larøi and Woodward have suggested that AVH could result from combinatoric abnormalities in inner space-outer space and self-other distinctions, where either can be present or absent (Larøi and Woodward, 2007). We have investigated these capacities in the same subjects and with similar experimental designs and found, as has been previously reported, that hallucinating schizophrenia patients showed other- and outer space-misattributions (Stephane et al., 2010a,c). More importantly, we found that inner space-outer space and self-other distinction capacities are independent (Stephane et al., in preparation), which mirrors nicely the different “where” and “what” pathways for speech processing (Badcock, 2010). Recently, Waters et al. (2012), presented the most elaborate model yet, which combines multiple deficits/abnormal activity including the auditory cortex, signal detection, intentional inhibition, and top-down factors. According to my proposal above, all deficits need not be present in all subjects. Any given deficit can be present or absent in any given subject, which makes hallucinating subjects different from each other as is commonly observed.

Finally, the current proposal is meant as a framework for resolving the puzzle of AVH and not a resolution for the puzzle itself. It has two main practical implications for future research.

First, taking in consideration AVH phenomenology (i.e., studying subgroups phenomenologically defined) could improve the signal/noise ratio. If indeed the neural basis of AVH differs in specific aspects according to AVH subtypes (e.g., inside or outside the head location of AVH), an experimental design that include both subtypes in one group would add noise to the signal generated by the aspect that differentiate the two subtypes. Thus, this approach could maximize the chance of understanding of the cognitive and neural basis of AVH. Better understanding of the cognitive basis could also facilitate treatment efforts for AVH through cognitive remediation.

Second, the phenomenological subgroups could also inform the experimental design. Any given AVH characteristic could provide clues about the malfunctioning cognitive and neural processes that resulted in AVH with that particular characteristic. For example AVH with lexical, sentencial, and discourse linguistic complexity could point to dysfunction in lexical, sentential, and discourse processes, respectively. Therefore, the appropriate cognitive task could be designed for the appropriate phenomenological type.

Finally, the success of phenomenological research depends in no small measures on the reliable identification of the phenomenological subtypes.

### Conflict of interest statement

The author declares that the research was conducted in the absence of any commercial or financial relationships that could be construed as a potential conflict of interest.
